# Supplementary material for: Multiple ParA/MinD ATPases coordinate the positioning of disparate cargos in a bacterial cell
Source: Nat Commun. 2023 Jun 5;14:3255. doi: 10.1038/s41467-023-39019-x (PMC10241942; doi:10.1038/s41467-023-39019-x)
Supplement: Supplementary file 1 — Supplementary Information [file 41467_2023_39019_MOESM1_ESM.pdf]

Multiple ParA/MinD ATPases coordinate the positioning of disparate cargos in a bacterial cell

Lisa T. Pulianmackal[1], Jose Miguel I. Limcaoco[2], Keerthikka Ravi[3], Sinyu Yang[3], Jeffrey Zhang[3], Mimi K. Tran[3], Maria Ghalmi[3], Matthew J. O'Meara[2] & Anthony G. Vecchiarelli[3*]

Supplementary information

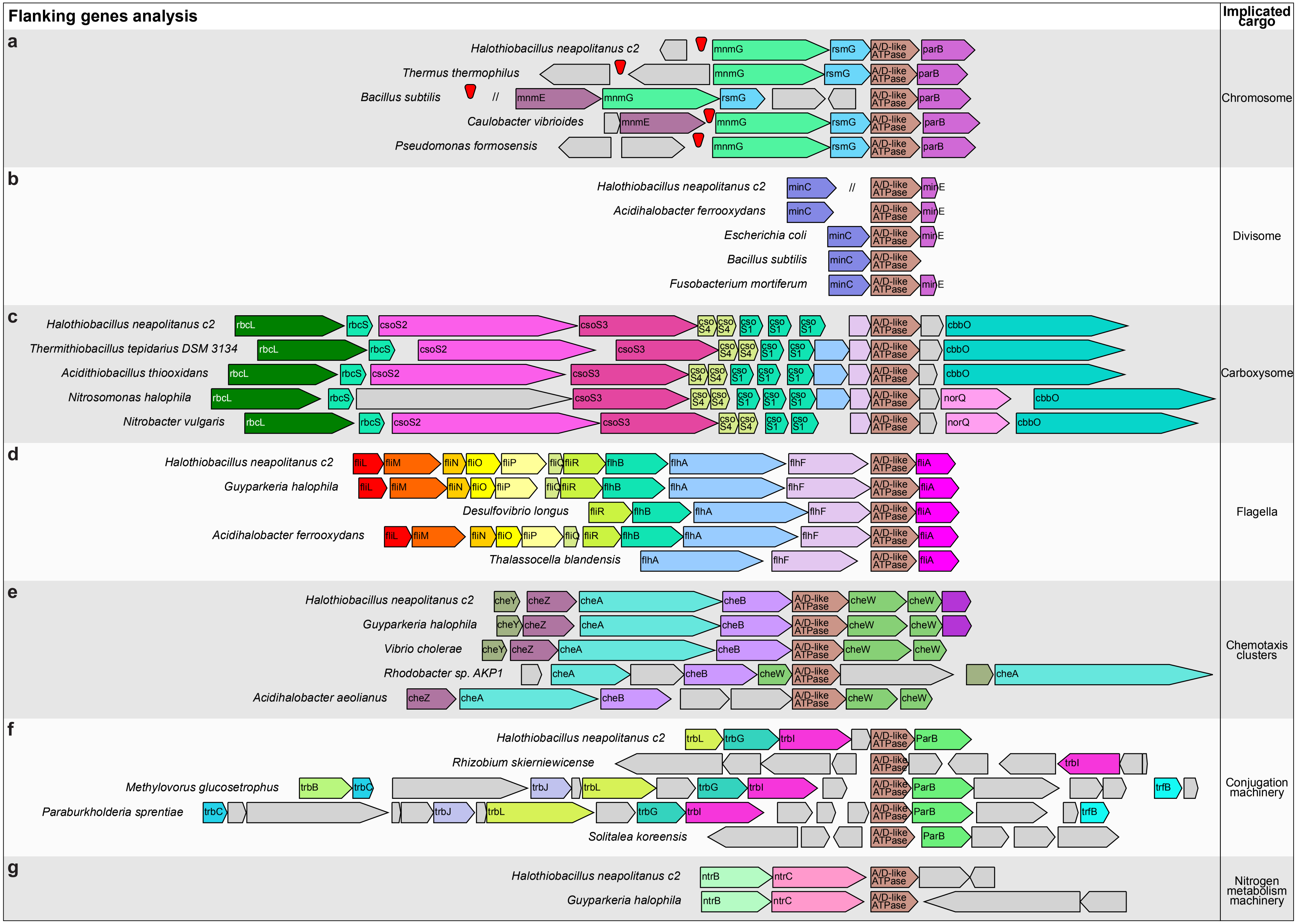
Flanking genes analysis
Implicated cargo
a
Halothiobacillus neapolitanus c2
Thermus thermophilus
Bacillus subtilis
Caulobacter vibrioides
Pseudomonas formosensis
mnmE
mnmG
rsmG
A/D-like ATPase
parB
Chromosome
b
Halothiobacillus neapolitanus c2
Acidihalobacter ferrooxydans
Escherichia coli
Bacillus subtilis
Fusobacterium mortiferum
minC
minE
Divisome
c
Halothiobacillus neapolitanus c2
Thermithiobacillus tepidarius DSM 3134
Acidithiobacillus thiooxidans
Nitrosomonas halophila
Nitrobacter vulgaris
rbcL
rbcS
csoS2
csoS3
cso S4
cso S1
norQ
cbbO
Carboxysome
d
Halothiobacillus neapolitanus c2
Guyparkeria halophila
Desulfovibrio longus
Acidihalobacter ferrooxydans
Thalassocella blandensis
fliL
fliM
fliN
fliO
fliP
fliQ
fliR
flhB
flhA
flhF
fliA
Flagella
e
Halothiobacillus neapolitanus c2
Guyparkeria halophila
Vibrio cholerae
Rhodobacter sp. AKP1
Acidihalobacter aeolianus
cheY
cheZ
cheA
cheB
cheW
Chemotaxis clusters
f
Halothiobacillus neapolitanus c2
Rhizobium skierniewicense
Methylovorus glucosetrophus
Paraburkholderia sprentiae
Solitalea koreensis
trbB
trbC
trbJ
trbL
trbG
trbI
ParB
trfB
Conjugation machinery
g
Halothiobacillus neapolitanus c2
Guyparkeria halophila
ntrB
ntrC
Nitrogen metabolism machinery

Supplementary Figure 1: Flanking Genes (FlaGs) analysis shows conservation among A/D ATPase gene neighborhoods. a The A/D ATPase gene involved in chromosome segregation (*parA*) is typically found a few genes downstream of the origin of replication (red cone) and upstream of a *parB* gene. **b** The A/D ATPase gene involved in divisome positioning (*minD*) is typically between genes encoding MinC and MinE. *minC* in *H. neapolitanus* was found elsewhere in the genome. **c** The A/D ATPase gene involved in carboxysome distribution (*mcdA*) is typically found near carboxysome shell components. **d** The A/D ATPase gene involved in flagella positioning (*flhG*) is typically found near core components of the flagellar apparatus. **e** The A/D ATPase gene involved in chemotaxis cluster positioning (*parC*) is typically found near genes required for chemotaxis cluster formation and regulation. **f** The gene neighborhoods of conjugation operons are not well conserved. However, the A/D ATPase gene involved in spatially organizing conjugation (*virC1*) is typically found upstream of a ParB-like protein. The conjugation operons in *Methylovorus glucosetrophus* and *Paraburkholderia sprentiae* were found on plasmids pMsip01 and pI3WSM5005, respectively. **g** Gene neighborhood of A/D ATPase genes associated with nitrogen metabolism are not well conserved. Only one other organism had similar neighboring genes as *H. neapolitanus*.

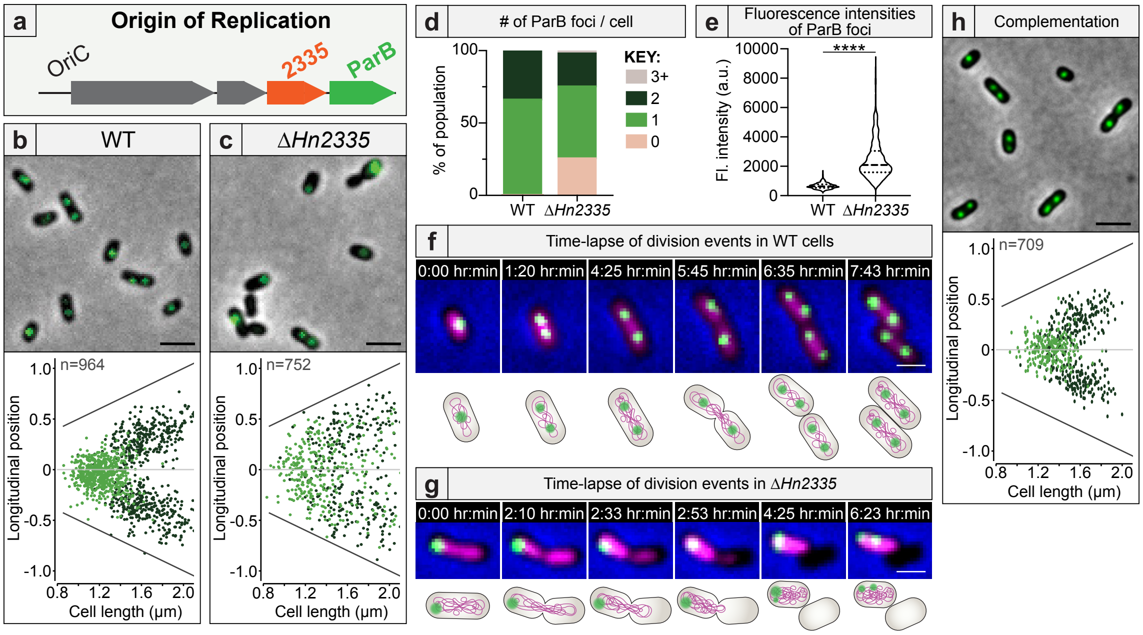

Supplementary Figure 2: *Hn2335* is required for chromosome segregation in *H. neapolitanus*. a *Hn2335* is found near the origin of replication (*OriC*) and has a ParB-homolog encoded immediately downstream. The genomic location of *Hn2335* suggests it encodes for the chromosome segregation ParA ATPase. **b** The chromosome origin of replication was tagged by labelling the ParB homolog with mNeonGreen to form ParB-mNG. Population analysis of foci localization: Cells were analyzed and quantified using MicrobeJ. On the x-axis, cells are organized according to increasing cell length. The y-axis represents the distance from mid-cell (µm). The foci on the graphs represent where the ParB foci are found along the length of the cell. Light green: 1 focus/cell; dark green: 2 foci/cell. Short WT cells had a single ParB focus at mid-cell, whereas longer cells had two foci at the quarter positions. Scale bar: 2 µm **c** Δ*Hn2335* mutant cells displayed random positioning of ParB foci regardless of cell length. Scale bar: 2 µm **d** WT cells had 1-2 foci. 25% of Δ*Hn2335* cells had no foci. **e** Δ*Hn2335* cells had much brighter ParB foci compared to WT. WT: n = 964; Δ*Hn2335*: n = 752 biologically independent cells. Two-sided Wilcoxon test p-value < 0.0001 **f** Newborn WT cells have a single ParB focus at mid-cell. Foci are then faithfully segregated to the quarter positions prior to division. Green: ParB foci; Magenta: SytoxBlue stain. Scale bar: 1 µm **g** In Δ*Hn2335*, faithful foci positioning and segregation is lost. Because both ParB foci remain on the left-hand side of the dividing cell, the cell on the right becomes anucleate following cell division. Green: ParB foci; Magenta: SytoxBlue nucleoid stain. Scale bar: 1 µm **h** Exogenous expression of *Hn2335* restored ParB foci positioning. Cells were induced with 50 µM IPTG for 3 hours. Scale bar: 2 µm (Panels a-c are duplicated from Figure 2.)

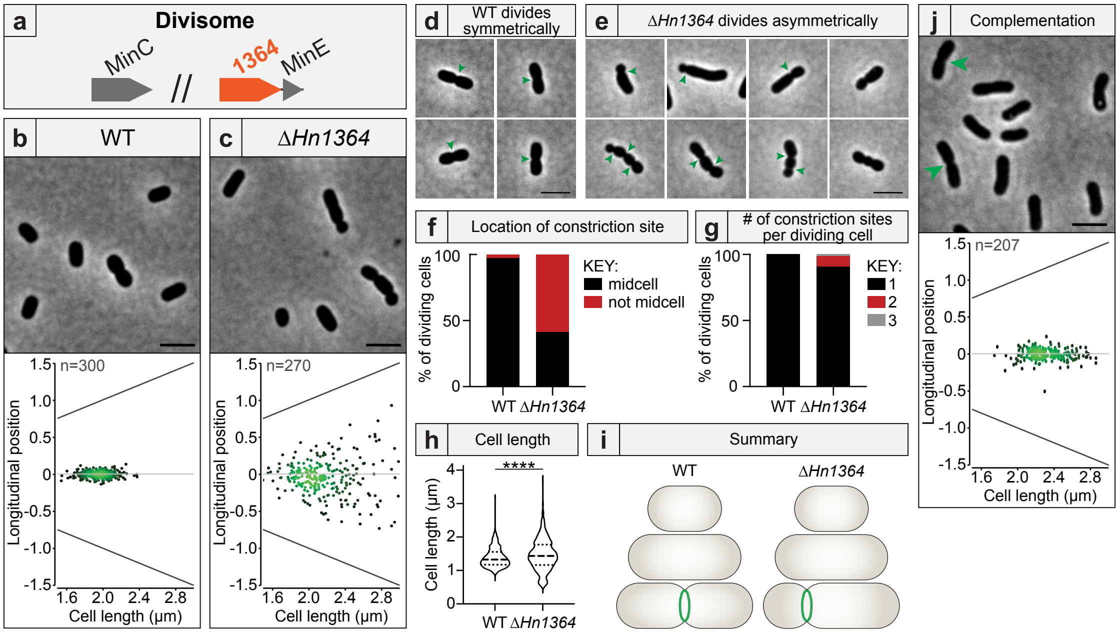

Supplementary Figure 3: *Hn1364* is required for cell division positioning. a *Hn1364* is found directly upstream of *minE*. *minC* is also present elsewhere in the genome. Therefore, the MinCDE system is present and likely involved in divisome positioning in *H. neapolitanus*. **b, c** Divisome positioning was determined by the location of constriction sites. Cells were analyzed and quantified using MicrobeJ. For each mutant, ~300 dividing cells were analyzed for the location of their constriction sites relative to mid-cell. On the x-axis, cells are organized according to increasing cell length. The y-axis represents the distance from mid-cell (µm). Each dot on the graph represents one identified constriction site. In this density plot, light green represents high density and dark green represents low density. In WT cells, constriction sites were found close to mid-cell. In Δ*Hn1364*, constriction sites could be found throughout the length of the cell. Scale bar: 2 µm **d** WT cells had constriction sites at mid-cell (green arrows). Scale bar: 2 µm **e** Δ*Hn1364* cells were more likely to divide asymmetrically at non-mid-cell locations (green arrows). Multiple division sites could also be found simultaneously on the same cell. Scale bar: 2 µm **f** 97% of WT cells divided at mid-cell. Only 41% of Δ*Hn1364* cells divided at mid-cell. Constriction sites were considered “mid-cell” when they were found within 5% of the cell center along the long axis. **g** WT cells only had one division site per cell at any given time. In Δ*Hn1364*, 9% of dividing cells had multiple division sites per cell. **h** Mutant cells displayed greater variability in cell size. WT: n = 3721; Δ*Hn1364*: n = 1780 biologically independent cells. Two-sided Wilcoxon test p-value < 0.0001 **i** *Hn1364* is critical for positioning the divisome at mid-cell in *H. neapolitanus*. **j** Exogenous expression of *Hn1364* restored mid-cell constriction. Prior to induction, 38% of cells divided at mid-cell. Induction with 50 µM IPTG for 6 hours increased the proportion of mid-cell divisions to 87%. Additionally, only 2% of dividing cells had multiple division cites per cell. Scale bar: 2 µm (Panels a-c are duplicated from Figure 2.)

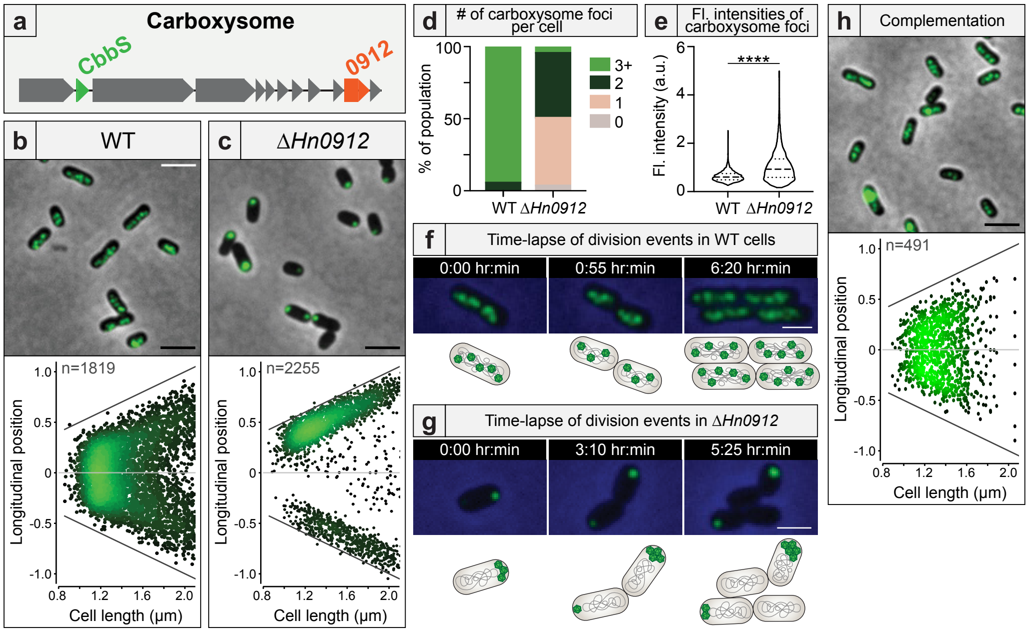

Supplementary Figure 4: Carboxysome positioning is determined by McdA, the ParA/MinD-like ATPase encoded in the carboxysome operon. a *Hn0912* encodes McdA and is found near genes encoding carboxysome shell proteins and RuBisCO. **b** Carboxysomes were visualized by labelling the small subunit of the Rubisco enzyme (*cbbS*) with mTurquoise2 to form CbbS-mTQ. Population analysis of foci localization: Cells were analyzed and quantified using MicrobeJ. On the x-axis, cells are organized according to increasing cell length. The y-axis represents the distance from mid-cell (µm). The foci on the graphs represent where the foci are found along the length of the cell. In WT cells, carboxysomes are distributed across the cell length. Scale bar: 2 µm **c** In Δ*mcdA*, carboxysomes formed a large polar focus at one or both poles. The pole of the cell that is closest to a focus is oriented to the top. Foci in the bottom half of the graph indicate a second focus. Scale bar: 2 µm **d** Carboxysome foci count varied dramatically between WT and mutant. In WT, 95% of cells had three or more foci, compared to only 5% of mutant cells. Instead, the vast majority of mutant cells had one or two carboxysome foci at the cell poles. Additionally, ~ 5% of the mutant population had no foci, which was never observed in WT cells. **e** Fluorescence intensity analysis of the foci revealed that, although the mutant population had fewer foci, the foci were significantly brighter. WT: n = 1819; Δ*Hn0912*: n = 2255 biologically independent cells. Two-sided Wilcoxon test p-value < 0.0001 **f** In WT, carboxysomes are dynamically positioned along the cell length throughout the cell cycle and across multiple generations. Scale bar: 2 µm **g** In Δ*mcdA*, carboxysome aggregates were stagnant throughout the cell cycle and across multiple generations. Scale bar: 2 µm **h** Exogenous expression of *Hn0911-Hn0912* restored carboxysome positioning. Prior to induction, carboxysome aggregates were found at the poles. Induction with 50 µM IPTG for 3 hours resulted in re-positioning of carboxysomes over the nucleoid and along the cell length. Scale bar: 2 µm (Panels a-c are duplicated from Figure 2.)

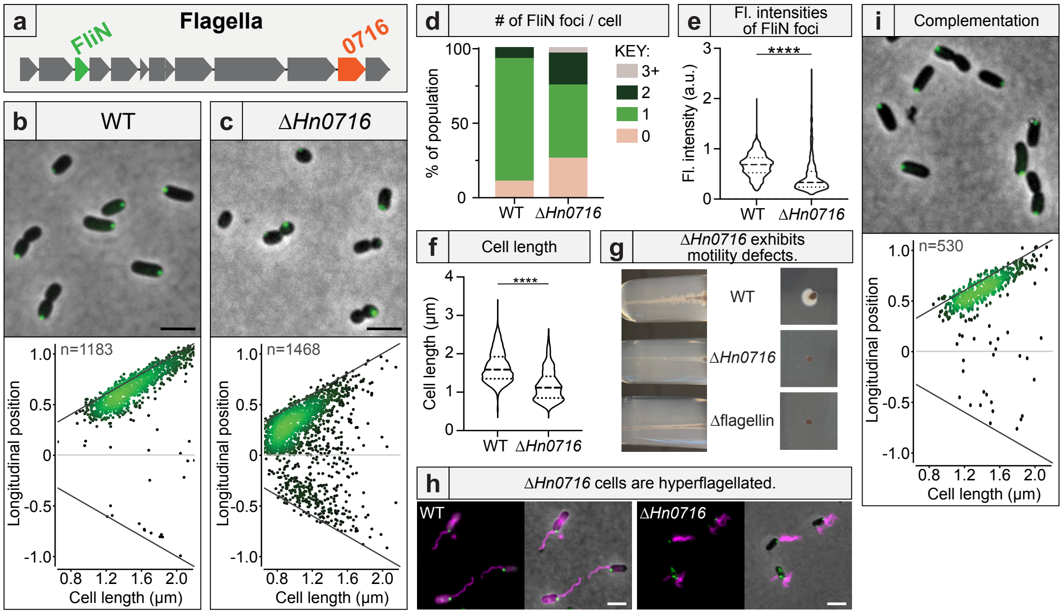

Supplementary Figure 5: *Hn0716* is required for regulating flagella position and copy number. a *Hn0716* is found near flagella-associated genes, suggesting it is involved in positioning flagella. **b** Flagella localization was visualized by labelling a component of the cytoplasmic ring of the flagellar basal body (*fliN*) with mNeonGreen to form mNG-FliN. Population analysis of foci localization: Cells were analyzed and quantified using MicrobeJ. On the x-axis, cells are organized according to increasing cell length. The foci on the graphs represent where the foci are found along the length of the cell. The pole of the cell that is closest to the FliN focus is oriented to the top. Foci under the mid-cell mark represent a second focus in the cell. WT cells typically had a single polar FliN focus. Scale bar: 2 µm **c** In Δ*Hn0716* cells, FliN foci were more randomly positioned along the cell length. Scale bar: 2 µm **d** 85% of WT cells had a single focus whereas only 45% of Δ*Hn0716* cells had a single focus. Also, instead of having a single polar focus, mutant cells were 3.5 times more likely to have two or more foci and 2.5 times more likely to have no foci at all. **e** When FliN foci were present in Δ*Hn0716* cells, the foci were much dimmer than those of WT cells. WT: n = 1183; Δ*Hn0716*: n = 1468 biologically independent cells. Two-sided Wilcoxon test p-value < 0.0001 **f** Δ*Hn0716* cells were shorter than WT cells. WT: n = 1183; Δ*Hn0716*: n = 1468 biologically independent cells. Two-sided Wilcoxon test p-value < 0.0001 **g** Δ*Hn0716* was not motile in motility assays. **h** WT cells had a single polar flagellum next to the FliN focus. Δ*Hn0716* cells typically had multiple flagella, often as tufts, emanating from multiple FliN foci. Micrographs shown are representative images from 2 experiments. Scale bar: 2 µm **i** Exogenous expression of *Hn0716* restored FliN localization to the poles. Cells were not induced with IPTG. Instead, leaky expression was sufficient for a recovery. Scale bar: 2 µm (Panels a-c are duplicated from Figure 2.)

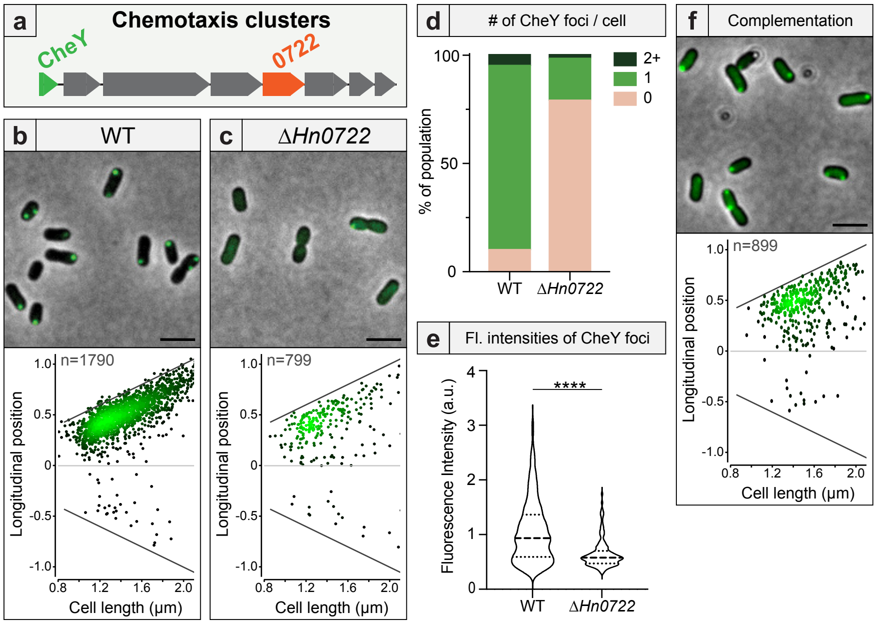

Supplementary Figure 6: *Hn0722* is required for chemotaxis cluster positioning. a *Hn0722* is found near chemotaxis-associated genes, suggesting its involvement in positioning chemotaxis clusters. **b** Chemotaxis clusters were visualized by labelling the response regulator (*cheY*) with mNeonGreen to form CheY-mNG. Population analysis of foci localization: Cells were analyzed and quantified using MicrobeJ. On the x-axis, cells are organized according to increasing cell length. The foci on the graphs represent where the foci are found along the length of the cell. The pole of the cell that is closest to the CheY focus is oriented to the top. Foci under the mid-cell mark represent a second focus in the cell. WT cells had a single CheY polar focus. Scale bar: 2 µm **c** Δ*Hn0722* mutant cells typically had no CheY foci. Scale bar: 2 µm **d** 87% of WT cells had one focus whereas only 19% of Δ*Hn0722* cells had a single focus. **e** When Δ*Hn0722* cells did have a detectable focus, the foci were much dimmer in fluorescence intensity compared to WT. WT: n = 1790; Δ*Hn0722*: n = 799 biologically independent cells. Two-sided Wilcoxon test p-value < 0.0001 **f** Exogenous expression of *Hn0722-Hn0723* restored CheY foci at the poles. Cells were induced with 50 µM IPTG for 3 hours. Scale bar: 2 µm (Panels a-c are duplicated from Figure 2.)

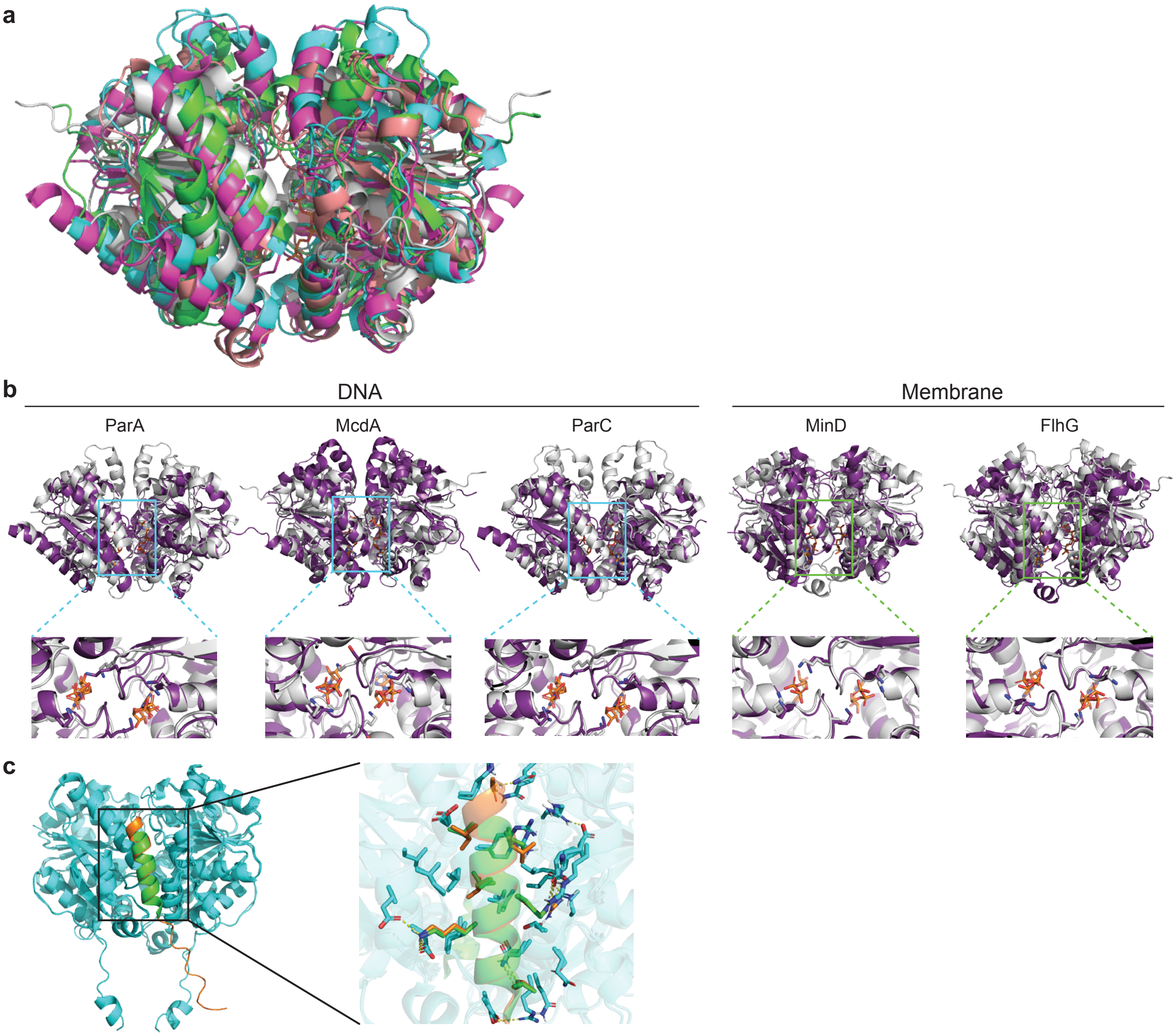

Supplementary Figure 7: AlphaFold2 structural predictions for the A/D ATPases of *H. neapolitanus*. a AlphaFold2 predicted models of A/D ATPases in *H. neapolitanus* overlaid showing similar sandwich homodimers. ParA (magenta), McdA (gray), ParC (cyan), MinD (salmon), and FlhG (green). **b** Predicted A/D ATPase structures from *H. neapolitanus* (purple) overlaid with experimentally determined homologs (gray) from the Protein Data Bank (PDB). ParA, McdA, ParC, MinD, and FlhG were overlaid with PDB ID #'s 5U1G, 6NOP, 5U1G 3QPL, and 4R23 respectively. ATP-binding pockets are zoomed in below - the indicated signature lysine defines the A/D ATPase family and interacts with the γ-phosphate of the ATP molecule (orange) bound to the opposing monomer. **c** *In silico* alanine-substitution simulations identify the *H. neapolitanus* MinE residues (orange) experimentally shown to be important for docking the *Escherichia coli* MinE peptide (green) onto the MinD dimer in cyan (PDB ID 3Q9L). Predicted *H. neapolitanus* MinD dimer is shown overlaid in cyan.

Supplementary Discussion

Deletion of *flhG* in *H. neapolitanus* resulted in a novel phenotype. The role of FlhG has been primarily investigated in polar flagellates of the gammaproteobacterial class; particularly *Pseudomonas*, *Shewanella*, and *Vibrio* species [1–7]. In these organisms, deletion of *flhG* results in hyper-flagellation and decreased motility. In other organisms, there are key differences in the mutant phenotype. For example, in *Bacillus subtilis*, *flhG* deletion results in tufts of flagella, but motility is unaffected [8]. In *Helicobacter pylori*, flagella are completely lost [9]. Given that *H. neapolitanus* is a polar flagellate in the gammaproteobacterial class, we expected that a deletion of *flhG* would result in just hyper-flagellation. However, the phenotype in *H. neapolitanus* was pleiotropic, unlike the gammaproteobacteria previously investigated. Deletion of *flhG* resulted in, not only hyper-flagellation **(Supplementary Figure 5h)**, but surprisingly, flagella mislocalization **(Supplementary Figure 5c)**, cell division defects **(Supplementary Figure 5f)**, and dimmer FliN foci **(Supplementary Figure 5e)**.

In polar flagellates, deletion of the GTPase FlhF, not FlhG, results in flagella mislocalization[10]. The *flhF* gene is typically encoded immediately upstream of *flhG*. In *H. neapolitanus*, a putative *flhF* is upstream of *flhG*, but its role remains to be investigated. It is important to reiterate that expression of just *flhG* at an exogenous locus restored FliN localization and cell division defects (**Supplementary Figure 5i**).

In the unusual case of *Campylobacter jejuni*, deletion of *flhG* results in hyper-flagellation and decreased motility, but also cell division defects [11,12]. *C. jejuni* lacks a Min system for divisome positioning, so it was proposed that FlhG may fulfill this role. Unlike *C. jejuni*, however, *H. neapolitanus* has a Min system. Therefore, the link between *flhG* and cell division in *H. neapolitanus* remains unclear.

Finally, FliN is a component of the flagella basal body. Given the increase in flagella number in the Δ*flhG* mutant, we expected more flagella basal bodies and a corresponding increase in FliN focus intensity. On the contrary, FliN foci were dimmer **(Supplementary Figure 5e)**,

suggesting FlhG may also play a role in basal body assembly in *H. neapolitanus*. Future studies will investigate the pleiotropy of *flhG* in flagella assembly, number, and location, as well as cell division.

Supplementary References

1 Campos-García J, Nájera R, Camarena L, Soberón-Chávez G. The Pseudomonas aeruginosa motR gene involved in regulation of bacterial motility. *FEMS Microbiol Lett* 2000;**184**:57–62. https://doi.org/10.1111/J.1574-6968.2000.TB08990.X.

2 Dasgupta N, Arora SK, Ramphal R. fleN, a gene that regulates flagellar number in Pseudomonas aeruginosa. *J Bacteriol* 2000;**182**:357–64. https://doi.org/10.1128/JB.182.2.357-364.2000.

3 Schuhmacher JS, Rossmann F, Dempwolff F, Knauer C, Altegoer F, Steinchen W, *et al.* MinD-like ATPase FlhG effects location and number of bacterial flagella during C-ring assembly. *Proc Natl Acad Sci U S A* 2015;**112**:3092–7. https://doi.org/10.1073/PNAS.1419388112.

4 Kusumoto A, Shinohara A, Terashima H, Kojima S, Yakushi T, Homma M. Collaboration of FlhF and FlhG to regulate polar-flagella number and localization in Vibrio alginolyticus. *Microbiol* 2008;**154**:1390–9. https://doi.org/10.1099/mic.0.2007/012641-0.

5 Kusumoto A, Kamisaka K, Yakushi T, Terashima H, Shinohara A, Homma M. Regulation of polar flagellar number by the flhF and flhG genes in Vibrio alginolyticus. *J Biochem* 2006;**139**:113–21. https://doi.org/10.1093/JB/MVJ010.

6 Arroyo-Pérez EE, Ringgaard S. Interdependent Polar Localization of FlhF and FlhG and Their Importance for Flagellum Formation of Vibrio parahaemolyticus. *Front Microbiol* 2021;**0**:557. https://doi.org/10.3389/FMICB.2021.655239.

7 Gao T, Shi M, Ju L, Gao H. Investigation into FlhFG reveals distinct features of FlhF in regulating flagellum polarity in Shewanella oneidensis. *Mol Microbiol* 2015;**98**:571–85. https://doi.org/https://doi.org/10.1111/mmi.13141.

8 Guttenplan SB, Shaw S, Kearns DB. The cell biology of peritrichous flagella in Bacillus subtilis. *Mol Microbiol* 2013;**87**:211–29. https://doi.org/https://doi.org/10.1111/mmi.12103.

9 Amsterdam K Van, Ende A Van Der. Helicobacter pylori HP1034 (ylxH) is required for motility. *Helicobacter* 2004;**9**:387–95. https://doi.org/10.1111/J.1083-4389.2004.00268.X.

10 Kondo S, Imura Y, Mizuno A, Homma M, Kojima S. Biochemical analysis of GTPase FlhF which controls the number and position of flagellar formation in marine Vibrio. *Sci Rep* 2018;**8**:. https://doi.org/10.1038/s41598-018-30531-5.

11 Gulbronson CJ, Ribardo DA, Balaban M, Knauer C, Bange G, Hendrixson DR. FlhG employs diverse intrinsic domains and influences FlhF GTPase activity to numerically regulate polar flagellar biogenesis in Campylobacter jejuni. *Mol Microbiol* 2016;**99**:291–306. https://doi.org/10.1111/mmi.13231.

12 Balaban M, Hendrixson DR. Polar Flagellar Biosynthesis and a Regulator of Flagellar Number Influence Spatial Parameters of Cell Division in Campylobacter jejuni. *PLOS Pathog* 2011;**7**:e1002420.
