# Supplementary material for: Multiple ParA/MinD ATPases coordinate the positioning of disparate cargos in a bacterial cell
Source: Nat Commun. 2023 Jun 5;14:3255. doi: 10.1038/s41467-023-39019-x (PMC10241942; doi:10.1038/s41467-023-39019-x)
Supplement: Supplementary file 3 — Description of Additional Supplementary Files [file 41467_2023_39019_MOESM3_ESM.pdf]

Description of Additional Supplementary Files:

Supplementary Data 1: ParA/MinD family ATPase hits across the bacterial domain binned by bacterial specie.

Supplementary Data 2: ParA/MinD ATPase distribution and predicted type from the NCBI RefSeq Database. Bacteria with no A/D ATPase hits are also provided.

Supplementary Data 3: Amino acids comprising the interfaces of ParA/MinD family ATPases in *H.neapolitanus* predicted to be important specificity determinants for homodimerization (Tab 1), positioning matrix association (Tab 2), and partner protein binding (Tab 3). Tab 4 shows in silico alanine-substitution mutagenesis and the resulting ΔΔG values for all residues comprising the N-terminal peptides of partner proteins docked onto their cognate A/D ATPase.

Supplementary Movie 1: Hn2335 is required for chromosome segregation in *H. neapolitanus*. (A) Time-lapse microscopy of ParB-mNG foci (green) and SYTOX-stained nucleoids (magenta) showing newborn WT cells with a single ParB focus at mid-cell, which then splits into two foci that bidirectionally segregate towards the quarter positions of the growing cell. Foci positioning at the quarters of the cell was maintained, which then became the mid-cell position of each daughter cell following division. (B) In the ΔHn2335 (ΔparA) mutant, faithful chromosome segregation and inheritance were lost, resulting in polyploid cells that continued to divide and non-viable anucleate cells. Arrow highlights the invaginationdependent spooling of the chromosome immediately prior to complete septation. Phase Contrast (blue) shows cell perimeter. Videos accelerated ~2,400 x real time.

Supplementary Movie 2: Hn1364 is required for cell division positioning. (A) Time-lapse microscopy shows WT cells dividing at mid-cell. (B) ΔHn1364 (ΔminD) cells divide asymmetrically. (C) ΔminD cells occasionally undergo multiple divisions simultaneously along the cell length. Phase Contrast (blue) shows cell perimeter. Arrows highlight division sites. Videos accelerated ~2,400 x real time in (A, B) and ~19,000 x real time in (C).

Supplementary Movie 3: Carboxysome positioning is determined by McdA, the A/D ATPase encoded in the carboxysome operon. (A) Time-lapse microscopy of fluorescent-labelled carboxysomes (green) in dividing WT cells shows that carboxysomes are dynamically positioned along the cell length throughout the cell cycle and across multiple generations. (B) In ΔHn0912 (ΔmcdA) cells, carboxysome aggregates were static at the cell poles throughout the cell cycle and across multiple generations. Phase Contrast (blue) shows cell perimeter. Videos accelerated ~2,400 x real time.

Supplementary Movie 4: Chromosome positioning is still actively maintained in ΔflhG. Time-lapse microscopy of ParB-mNG foci (green) in a ΔflhG mutant shows that chromosome segregation remains functional. But, anucleate cells form because longer cells only have a single chromosome, suggesting a defect in chromosome replication. Phase Contrast (blue) shows cell perimeter. Video accelerated ~2,400 x real time.

Supplementary Movie 5: Carboxysomes are inherited in anucleate cells. Time-lapse microscopy of fluorescent-labelled carboxysomes (green) in (A) ΔparA, (B) ΔminD, and (C) ΔflhG strains showing that carboxysomes can be inherited in anucleate cells. Carboxysomes in the to-be-anucleate cell bundled up immediately adjacent to the division plane (green arrows). Carboxysome bundling was coincident with chromosome extrusion through the invaginating septum just prior to complete division and asymmetric chromosome inheritance. After septation, the carboxysome bundle was explosively liberated from the anucleate cell pole, resulting in multiple freely diffusible carboxysome foci. Anucleate cells harboring carboxysomes did not divide further. Phase Contrast (blue) shows cell perimeter. Videos accelerated ~2,400 x real time.
