# Supplementary material for: Multiple ParA/MinD ATPases coordinate the positioning of disparate cargos in a bacterial cell
Source: Nat Commun. 2023 Jun 5;14:3255. doi: 10.1038/s41467-023-39019-x (PMC10241942; doi:10.1038/s41467-023-39019-x)
Supplement: Supplementary file 12 — Reporting Summary [file 41467_2023_39019_MOESM12_ESM.pdf]

nature portfolio

Corresponding author(s): Anthony G. Vecchiarelli

Last updated by author(s): May 15, 2023

Reporting Summary

Nature Portfolio wishes to improve the reproducibility of the work that we publish. This form provides structure for consistency and transparency in reporting. For further information on Nature Portfolio policies, see our Editorial Policies and the Editorial Policy Checklist.

Statistics

For all statistical analyses, confirm that the following items are present in the figure legend, table legend, main text, or Methods section.

n/a	Confirmed	
☐	☒	The exact sample size (n) for each experimental group/condition, given as a discrete number and unit of measurement
☐	☒	A statement on whether measurements were taken from distinct samples or whether the same sample was measured repeatedly
☐	☒	The statistical test(s) used AND whether they are one- or two-sided<br>*Only common tests should be described solely by name; describe more complex techniques in the Methods section.*
☒	☐	A description of all covariates tested
☐	☒	A description of any assumptions or corrections, such as tests of normality and adjustment for multiple comparisons
☒	☐	A full description of the statistical parameters including central tendency (e.g. means) or other basic estimates (e.g. regression coefficient) AND variation (e.g. standard deviation) or associated estimates of uncertainty (e.g. confidence intervals)
☐	☒	For null hypothesis testing, the test statistic (e.g. F, t, r) with confidence intervals, effect sizes, degrees of freedom and P value noted<br>*Give P values as exact values whenever suitable.*
☒	☐	For Bayesian analysis, information on the choice of priors and Markov chain Monte Carlo settings
☒	☐	For hierarchical and complex designs, identification of the appropriate level for tests and full reporting of outcomes
☒	☐	Estimates of effect sizes (e.g. Cohen's d, Pearson's r), indicating how they were calculated

Our web collection on statistics for biologists contains articles on many of the points above.

Software and code

Policy information about availability of computer code

Data collection	NIS Elements AR 5.02.01 software was used for image acquisition. Protein structures were generated with the CollabFold implementation of AlphaFold2 (Mirdita et al., 2022; Jumper et al., 2021). Peptide docking models were generated using Rosetta's FlexPepDock protocol (Raveh et al., 2011). See methods for details.<br><br>The code generated during this study are available at GitHub:<br><br>Identifying ParA/MinD ATPases in bacteria using BLAST:<br>https://github.com/krthkkrv/Multiple-ParA-MinD-ATPases-coordinate-the-positioning-of-disparate-cargos-in-a-bacterial-cell.<br><br>Identifying specificity determinants of each ParA/MinD ATPase in H. neapolitanus using AlphaFold2 and Rosetta:<br>https://github.com/jilimcaoco/Multiple-ParA-MinD-ATPases.
Data analysis	All image analysis including cell identification, cell perimeter, quantification of cell length, foci localization, foci number, foci fluorescence intensity, and identification of constriction sites were performed using Fiji plugin MicrobeJ 5.13I (Schindelin et al., 2012; Ducret et al., 2016). Fluorescence intensity graphs and foci number count graphs were made in GraphPad Prism Version 9 (GraphPad Software, San Diego, CA, www.graphpad.com).

For manuscripts utilizing custom algorithms or software that are central to the research but not yet described in published literature, software must be made available to editors and reviewers. We strongly encourage code deposition in a community repository (e.g. GitHub). See the Nature Portfolio guidelines for submitting code & software for further information.

Data

Policy information about availability of data

All manuscripts must include a data availability statement. This statement should provide the following information, where applicable:

- Accession codes, unique identifiers, or web links for publicly available datasets
- A description of any restrictions on data availability
- For clinical datasets or third party data, please ensure that the statement adheres to our policy

Bacterial genomes obtained from the NCBI RefSeq DataBase (https://www.ncbi.nlm.nih.gov/refseq/). Experimentally determined ParA/MinD ATPase structures obtained from the Protein Data Bank (PDB): ParA (5U1G - https://www.rcsb.org/structure/5u1g); McdA (6NOP - https://www.rcsb.org/structure/6NOP); ParC (5U1G - https://www.rcsb.org/structure/5U1G); MinD (3Q9L - https://www.rcsb.org/structure/3Q9L); FlhG (4RZ3 - https://www.rcsb.org/structure/4RZ3). All data generated or analyzed during this study are included in this published article and its supplementary information files.
Source data are also provided with this paper.

Human research participants

Policy information about studies involving human research participants and Sex and Gender in Research.

Reporting on sex and gender	n/a
Population characteristics	n/a
Recruitment	n/a
Ethics oversight	n/a

Note that full information on the approval of the study protocol must also be provided in the manuscript.

Field-specific reporting

Please select the one below that is the best fit for your research. If you are not sure, read the appropriate sections before making your selection.

☒ Life sciences ☐ Behavioural & social sciences ☐ Ecological, evolutionary & environmental sciences

For a reference copy of the document with all sections, see nature.com/documents/nr-reporting-summary-flat.pdf

Life sciences study design

All studies must disclose on these points even when the disclosure is negative.

Sample size	No sample size calculation was performed. Sample size was determined to achieve a confidence level of 95% that the real value is within ±5% of the measured value. To achieve this, all data sets analyze at least 200 cells for each strain.
Data exclusions	No data was excluded from publication.
Replication	Technical replication - Imaging of strains were performed on at least three Field of Views (FOVs) and on different agarose pads.<br>Biological replicates - At least two different cultures, grown on different days were performed. Replication of data as was successful.
Randomization	Strains were grown on different days and imaged in random order. All image analysis was automated.
Blinding	During image acquisition, blinding was achieved by using the automated stage to move to four random fields of view (FOVs) during image acquisition. All image analysis including cell identification, cell perimeter, quantification of cell length, foci localization, foci number, foci fluorescence intensity, and identification of constriction sites was automated.

Reporting for specific materials, systems and methods

We require information from authors about some types of materials, experimental systems and methods used in many studies. Here, indicate whether each material, system or method listed is relevant to your study. If you are not sure if a list item applies to your research, read the appropriate section before selecting a response.

Materials & experimental systems

n/a	Involved in the study
☒	☐ Antibodies
☒	☐ Eukaryotic cell lines
☒	☐ Palaeontology and archaeology
☒	☐ Animals and other organisms
☒	☐ Clinical data
☒	☐ Dual use research of concern

Methods

n/a	Involved in the study
☒	☐ ChIP-seq
☒	☐ Flow cytometry
☒	☐ MRI-based neuroimaging
